# Chronic care services and variation between Danish general practices: a nationwide cohort study

**DOI:** 10.3399/BJGP.2021.0419

**Published:** 2022-02-08

**Authors:** Anders Prior, Claus Høstrup Vestergaard, Anette Riisgaard Ribe, Annelli Sandbæk, Flemming Bro, Peter Vedsted, Susan Smith, Mogens Vestergaard, Morten Fenger-Grøn

**Affiliations:** Research Unit for General Practice, Aarhus; Department of Public Health, Aarhus University, Aarhus, Denmark.; Research Unit for General Practice, Aarhus, Denmark.; Research Unit for General Practice, Aarhus, Denmark.; Department of Public Health, Aarhus University, Aarhus; Steno Diabetes Center Aarhus, Aarhus University Hospital, Aarhus, Denmark.; Research Unit for General Practice, Aarhus; Department of Public Health, Aarhus University, Aarhus, Denmark.; Research Unit for General Practice, Aarhus, Denmark.; Department of General Practice, RCSI University of Medicine and Health Sciences, Dublin, Ireland.; Research Unit for General Practice, Aarhus; Department of Public Health, Aarhus University, Aarhus, Denmark.; Research Unit for General Practice, Aarhus, Denmark.

**Keywords:** chronic disease, cohort studies, general practitioners, health services research, primary health care

## Abstract

**Background:**

Little is known about variations in the provision of chronic care services in primary care.

**Aim:**

To describe the frequency of chronic care services provided by GPs and analyse the extent of non-random variation in service provision.

**Design and setting:**

Nationwide cohort study undertaken in Denmark using data from 2016.

**Method:**

Information on chronic care services was obtained from national health registers, including annual chronic care consultations, chronic care procedures, outreach home visits, and talk therapy. The associations between services provided, patient morbidity, and socioeconomic factors were estimated. Service variations were analysed, and excess variation related to practice-specific factors was estimated while accounting for random variation.

**Results:**

Chronic care provision was associated with increasing patient age, increasing number of long-term conditions, and indicators of low socioeconomic status. Variation across practices ranged from 1.4 to 128 times more than expected after adjusting for differences in patient population and random variation. Variation related to practice-specific factors was present for all the chronic care services that were investigated. Older patients with lower socioeconomic status and multimorbidity were clustered in practices with low propensity to provide certain chronic care services.

**Conclusion:**

Chronic care was provided to patients typically in need of health care, that is, older adults, those with multimorbidity, and those with low socioeconomic status, but service provision varied more than expected across practices. GPs provided slightly fewer chronic care services than expected in practices where many patients with multimorbidity and low socioeconomic status were clustered, suggesting inverse care law mechanisms.

## INTRODUCTION

Modern health care is challenged by a significant burden from long-term chronic conditions that accumulate over time and lead to the development of multiple chronic conditions, or multimorbidity. Increasing numbers of middle-aged and older people are living with multimorbidity, adding significantly to the need for chronic care.^1^^,^^2^ In the Danish healthcare system (Box 1),^3^^–^7 GPs provide most chronic care for long-term conditions at the community level. This model ensures a high degree of continuity of care, which is valued by patients and known to improve health outcomes.^8^^–^14

**Box 1.** The Danish healthcare system and chronic care services

In the Danish tax-funded universal healthcare system, all residents have free access to a GP for medical advice or referrals to the secondary healthcare system (gatekeeper system). Virtually all residents (98%) are listed with a specific general practice.^3^ The list of each practice comprises approximately 1600 patients per GP.^4^ At the time of data collection, approximately 30% of practices were solo practices, 45% consisted of two to three GPs, and 25% consisted of four GPs or more.^4^ GPs work as independent primary care contractors for the health authorities and are remunerated through a mix of per capita and fee-for-service payments.^5^ Remunerated services include, for example, daytime consultations, email consultations, telephone contacts, home visits, laboratory services, and specific chronic care services (see below). Invoices for the provided services are sent to the Regional Health Administration who perform automated checks. They also check if there are practice outliers regarding use of specific services and, if so, they contact the practice for an explanation or correction. Data on remunerated services, reported by the practices using specific service codes, are available from the Danish National Health Service Register at the level of practices.^6^ *GP chronic care services* *The annual chronic care consultation*: a consultation dedicated to a specific chronic condition; it can be provided once a year per condition (with a maximum of four conditions per year) and may include a patient health overview, medication review, lifestyle talk, and goal setting. The remuneration is approximately 2.5 times that of an ordinary consultation. *Chronic care procedures*: para-clinical measurements related to chronic care management, including blood glucose measurements, electrocardiograms, lung function tests, and home blood pressure measurements. *Outreach home visits*: home visits initiated by the GP for frail individuals aged >70 years to provide an overview of the health situation. *Talk therapy*: this encompasses psychotherapy provided by the GP in consecutive sessions (a maximum of seven sessions per year) for patients with mental health conditions.^7^

Chronic care provision may vary among GP practices. This could reflect differences in the patient population served, for example, morbidity level, patient age profiles, and socioeconomic status. However, it could also be related to practice-specific factors, reflected in the practice chronic care profile, that is, practice-level differences in capacity, affiliated clinical and non-clinical staff, geographical setting, access to specialised care, and GP treatment preferences. Although homogeneity in care provision does not ensure optimal care, heterogeneity, that is, variation that cannot be explained by differences in patient characteristics, reflects over-and/or undertreatment of a condition. This leads to either suboptimal quality of care or inefficiency from a health-economic perspective.^15^^–^17 Further, service provision variation originating from socioeconomic differences may exacerbate health inequalities.^18^^,^^19^ Yet, it is essential to acknowledge that some variation is expected solely as a result of randomness.

Variation in health care is well studied, for example, the Dartmouth Atlas.^20^ However, the evidence on variation in chronic care management in primary care remains limited.^8^^,^^21^^–^24 In this exploratory study, the association between patient characteristics and chronic care service provision is described and the extent of non-random variation related to the practice chronic care profile is analysed.

## METHOD

### Study population and setting

A register-based nationwide cohort study in Denmark was conducted. The study population included all Danish citizens aged ≥18 years listed with a general practice (Box 1) and residing in Denmark for at least 5 consecutive years. The cohort was followed from 1 January 2016 until death, emigration, or end-of-study on 31 December 2016, whichever came first. Practices with <500 listed patient-years were excluded from the study (*n* = 184) as these were considered abnormally small and could represent newly started or liquidated practices, or a special administrative unit. All Danish residents have a unique identification number that can be used to link individuals across administrative registers.^25^ Information on all patients was obtained from the Danish national health registers and used the Patient List Database to link patients to a specific general practice.^26^

**Table:** How this fits in

There has been limited examination of variation in chronic care provision between general practices. This study found that chronic care was provided to patients typically in need of health care, including people who are socioeconomically deprived. However, variation in the provision of chronic care services could not be explained by patient population characteristics or by randomness; variation related to practice-specific factors was present. Fewer chronic care services than expected were provided by the general practices with patients who were the most multimorbid and socioeconomically deprived, which could suggest potential inverse care law mechanisms.

### Outcomes

The main outcomes were chronic care services provided by GPs, that is, annual chronic care consultations, chronic care procedures, outreach home visits, and talk therapy (Box 1). Additionally, other general contacts with the GP were included that were not necessarily related to chronic care, that is, ordinary daytime consultations, email consultations, telephone contacts, and home visits (see codes in Supplementary Table S1).

### Covariates

The Danish Civil Registration System and Statistics Denmark provided information on the patients’ age, sex, ethnicity, cohabitation status, household income, working status, and educational attainment at the start of follow-up, and on emigration and vital status during follow-up (see Supplementary Table S2).^25^^,^^27^

The Danish Multimorbidity Index^28^ provided information on 39 long-term conditions, which allowed the authors to assess disease and multimorbidity status (defined as ≥2 long-term conditions) for the patients (Supplementary Table S3).^28^^,^^29^

### Statistical analyses

First, multivariate Poisson models were constructed for the prediction of the number of each of the service code outcomes based on time-at-risk and baseline covariate status for each patient in the population. These models produced incidence rate ratios (IRRs). Second, the observed and the predicted number of outcomes for all patients listed with each practice were summed. Subsequently, these two aggregate measures were divided to obtain a practice-specific observed-to-expected (O/E) ratio expressing whether a practice provided more (O/E>1) or fewer (O/E<1) services than expected given the composition of the patient population. The practice O/E ratios were ranked and presented graphically. To quantify the amount of variation in the O/E ratios, the interdecile ratio (IDR) was used (that is, the 90th percentile divided by the 10th percentile). The IDR is a flexible measure that does not require assumptions regarding the distribution of the data, and it focuses on the high and low ends of the variation curve. The IDR is an actual factor of difference in provision of service between the practices and may be more easily interpreted for the average reader than, for example, standard deviations. For example, an IDR of 10 would mean that the practice representing the 90th percentile provided 10 times as many services as the practice representing the 10th percentile (adjusted for patient characteristics).

As a measure of the variation related to the chronic care profile of the individual general practices, the excess variation was calculated; this was done by dividing the observed IDR by a reference IDR corresponding to the variation expected because of chance alone (that is, if no true differences existed between practice profiles). This reference IDR was calculated by using a sampled reference population of patients from other practices (matched by sex, age, and a propensity score based on the remaining covariates).^30^

Third, to estimate the association between patient characteristics and the propensity for providing services in the individual practices, a series of Poisson regression models was constructed; one for each covariate–outcome combination. The inputs were the number of observed service code outcomes for each person’s practice, with a given covariate as the exposure, and the expected number of service code outcomes as the offset. Both the expected and the observed number of service code outcomes were analysed using a jack-knife type approach, that is, the contribution of each patient’s service code outcome was subtracted from the overall practice score.

To assess the correlation between annual chronic care consultations and general contacts, a Pearson coefficient between the two was calculated. All analyses were performed in Stata (version 16).

## RESULTS

In total, 1885 GP practices were identified, comprising a total patient population of 4.23 million patients aged ≥18 years (94% of the adult population). The practices had a median of approximately 2900 patients (interquartile range: 1700–4500). The demographic and socioeconomic characteristics of the study population are shown in Table 1 and details of their long-term health conditions are shown in Table 2.

**Table 1.** Demographic and socioeconomic characteristics of the study population

**Characteristic**	**Total population (*N* = 4 230 260), *n***	**%**
**Age, years**		
18–29	764 902	18.1
30–39	596 835	14.1
40–49	757 795	17.9
50–59	739 794	17.5
60–69	662 096	15.7
70–79	471 838	11.2
80–89	194 750	4.6
90–99	41 220	1.0
≥100	1030	0.02

**Sex**		
Female	2 154 965	50.9
Male	2 075 295	49.1

**Ethnicity**		
Danish	4 013 918	94.9
Western	99 365	2.3
Non-Western	116 977	2.8

**Cohabitation status**		
Cohabiting	2 719 951	64.3
Single	1 510 309	35.7

**Household income**		
1st quartile	1 007 447	23.8
2nd quartile	1 059 814	25.1
3rd quartile	1 053 596	24.9
4th quartile	1 109 403	26.2

**Working status**		
Employed	3 283 076	77.6
Unemployed	947 184	22.4

**Education, years**		
≤10	1 185 720	28.0
>10–≤15	1 990 605	47.1
>15	965 732	22.8
Unknown	88 203	2.1

**Table 2.** Study population long-term health conditions

**Characteristic**	**Total population (*N* = 4 230 260), *n***	**%**
Conditions, *n*		
0	2 210 240	52.2
1	835 410	19.7
2	474 951	11.2
3	304 977	7.2
≥4	404 682	9.6

**Specific conditions**	***n***	**%**

**Circulatory**		
Hypertension	823 918	19.5
Dyslipidaemia	406 320	9.6
Ischaemic heart disease	229 371	5.4
Atrial fibrillation	111 401	2.6
Heart failure	43 471	1.0
Peripheral artery occlusive disease	77 255	1.8
Stroke	115 669	2.7

**Endocrine**		
Diabetes mellitus	225 323	5.3
Thyroid disorder	148 985	3.5
Gout	19 021	0.4

**Pulmonary and allergic**		
Chronic pulmonary disease	244 465	5.8
Allergy	172 671	4.1

**Gastrointestinal**		
Ulcer/chronic gastritis	73 103	1.7
Chronic liver disease	33 127	0.8
Inflammatory bowel disease	47 741	1.1
Diverticular disease of intestine	75 352	1.8

**Urogenital**		
Chronic kidney disease	25 255	0.6
Prostate disorders	107 640	2.5

**Musculoskeletal**		
Connective tissue disorders	77 653	1.8
Osteoporosis	134 987	3.2
Painful condition	405 404	9.6

**Haematological**		
HIV/AIDS	3818	0.1
Anaemias	87 418	2.1

**Malignant conditions**		
Cancers	121 684	2.9

**Neurological**		
Vision problem	188 446	4.5
Hearing problem	207 163	4.9
Migraine	59 623	1.4
Epilepsy	38 723	0.9
Parkinson’s disease	6182	0.1
Multiple sclerosis	12 913	0.3
Neuropathies	34 943	0.8

**Mental health**		
Mood, stress-related, or anxiety disorders	9239	0.2
Psychological distress	306 093	7.2
Alcohol problems	7309	0.2
Substance abuse	1483	0.04
Anorexia/bulimia	444	0.01
Bipolar affective disorder	11 133	0.3
Schizophrenia or schizoaffective disorder	9969	0.2
Dementia	26 885	0.6

### GP service provision and patient characteristics

During follow-up, the practices provided an average of 16.7 annual chronic care consultations per 100 patient-years (Table 3). The rates of annual chronic care consultations and chronic care procedures were independently associated with patient age (peaking at 70–79 years), low income, and low educational level (Supplementary Figure S1). Most long-term conditions were associated with higher rates of annual chronic care consultations, for example, hypertension (IRR 2.24, 95% confidence interval [CI] = 2.23 to 2.26) and diabetes (IRR 1.50, 95% CI = 1.49 to 1.51), but with notable exceptions, for example, dementia (IRR 0.67, 95% CI = 0.65 to 0.68) and chronic kidney disease (IRR 0.68, 95% CI = 0.67 to 0.70).

**Table 3.** Chronic care provision — observed, reference, and excess variation

**GP service**	**Mean^b^**	**Observed variation**			**Reference (expectable) variation^a^**			**Excess variation^d^**
		**10th percentile**	**90th percentile**	**Adjusted IDR^c^**	**10th percentile**	**90th percentile**	**Adjusted IDR^c^**	
Annual chronic care consultations	16.65	0.18	1.74	9.95	0.92	1.07	1.17	8.54
Chronic care procedures^e^	33.76	0.03	2.21	8.41	0.64	1.32	1.17	7.21
Outreach home visits	2.55	0.21	1.75	79.55	0.91	1.07	2.07	38.48
Talk therapy	6.48	0.01	2.10	193.73	0.79	1.19	1.52	127.70
General contacts	752.01	0.80	1.23	1.53	0.96	1.03	1.07	1.42

a *Based on randomly sampled patients from other practices (matched by sex, age, and a propensity score based on the remaining covariates).*

b *Observed service outcomes per 100 patient-years (unadjusted).*

c *Adjusted for age, sex, ethnicity, cohabitation status, household income, working status, educational attainment, and 39 individual mental and physical long-term conditions.*

d *Excess variation (variation related to practice chronic care profile), that is, adjusted IDR/reference IDR.*

e *Blood glucose measurements, electrocardiograms, lung function tests, and home blood pressure measurements. IDR = interdecile ratio, that is, 90th percentile/10th percentile of the observed/expected ratio.*

Outreach visits were associated with older age, living alone, unemployment, and certain conditions, for example, dementia (IRR 4.00, 95% CI = 3.87 to 4.12). Talk therapy was associated with younger age, female sex, low income, most mental health conditions, and several physical conditions. Higher rates of general contacts were seen in older patients, female sex, and those with low income. General contacts were associated with almost all long-term conditions. All studied services were associated with the number of long-term conditions in a dose–response manner (Supplementary Table S4).

### Practice variation in service provision

**Figure 1.**
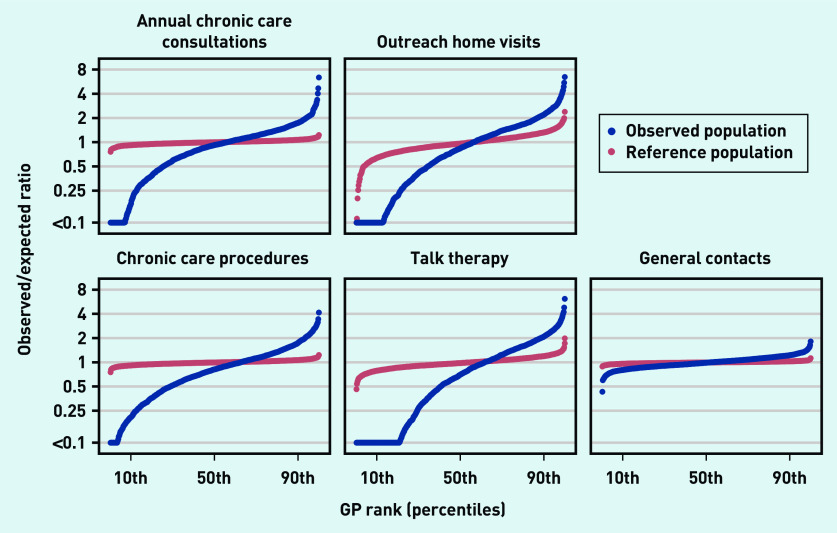
***Observed/expected ratios and reference variation in chronic care services across general practices.*** *^a^* *^a^* ***Blue curve: observed/expected ratios in chronic care services across practices adjusted for age, sex, ethnicity, cohabitation status, household income, working status, educational attainment, and 39 individual mental and physical long-term conditions. Red curve: observed/expected ratios in chronic care services across practices based on randomly sampled patients from other practices (matched by sex, age, and a propensity score based on the remaining covariates). Adjacent practices were arranged in groups of five on the plot to preserve anonymity.***

For all types of GP service code outcomes, the observed variation in the service provision across general practices was higher than expected based on the composition of the patient populations (Figure 1).

The magnitude of the variation, measured by the IDR, differed by GP service code outcome: the annual chronic care consultation was subject to an IDR of 9.95 across practices after adjustments (Table 3). The reference variation (owing to chance alone) was 1.17, resulting in an excess variation of 8.54. Chronic care procedures had an adjusted IDR of 8.41 with an excess variation of 7.21. The largest variation was found for outreach home visits and talk therapy. These varied by a factor of 80 and 194, respectively, with excess variations of 38 and 128, respectively, which was mainly because of very low implementation of these services in some practices. The general contacts showed an adjusted IDR of 1.53 and an excess variation of 1.42.

### GP service propensity and patient characteristics

Supplementary Figure S2 presents associations between patient characteristics and the propensity for service provision in general practices. Patients who were older, unemployed, less well educated, or had more long-term conditions were clustered in practices with a lower propensity to provide annual chronic care consultations and talk therapy, that is, practices that provided fewer than expected chronic care services based on their patient population. Conversely, the less well educated and patients with multimorbidity were clustered in practices with a high propensity to provide chronic care procedures. High education and high income were associated with a high propensity for GPs to provide outreach home visits. In comparison, patients who were older, unemployed, less well educated, and had multimorbidity were clustered in practices with an overall lower propensity to offer general contacts to their patients.

General practices with low propensity to provide annual chronic care consultations did not tend to compensate by offering more general contacts; a positive correlation was found between propensity for provision of annual chronic care consultations and general contacts (Pearson’s *r* = 0.11, *P*<0.001).

## DISCUSSION

### Summary

In this nationwide study, associations were found between chronic care services provided by Danish GPs and patient characteristics, such as old age, low education, low income, and high number of long-term conditions. However, the chronic care provision varied considerably across practices and the variation could not be explained by differences in the patient populations or by random variation. The excess variation related to the practice chronic care profile was seen for all types of services, ranging from 1.4 to 128 times more than expected. Furthermore, general practices with low propensity to provide certain chronic care services, including annual chronic care consultations, and general consultations had relatively more patients of an older age, low socioeconomic status, and multimorbidity.

The current study shows that GPs generally provide chronic care services to the patients in need of medical treatment. Patients with low socioeconomic status often have multimorbidity and a high disease burden. Thus, providing chronic care services may mitigate social inequality in health. This is in accordance with previous studies on healthcare utilisation in general^31^^–^33 and on primary care services in particular.^34^^,^^35^ In the current study, the GPs tended to conduct fewer chronic care services for some long-term conditions, which could reflect barriers at the patient level, or that the GPs might have considered the service redundant if chronic care was provided elsewhere, for example, in nursing homes for those with dementia or hospital out-patient clinics for those with chronic kidney disease.

The chronic care variation between general practice populations clearly exceeded the expected random variation (even after composition adjustment). The excess variation might be interpreted as an expression of the chronic care profile of the individual practice.^15^ This suggests that differences exist between the GPs in terms of knowledge, attitudes towards chronic care, experience, organisational skills, interpretation of guidelines, implementation of chronic care services, and remuneration coding practices. However, the treatment patterns may also be influenced by workload level, time constraints, the staffing levels of doctors and nurses, patient contact patterns, and local access to diagnostic tests and specialised health care.^16^

### Strength and limitations

The strengths of this study include the nationwide population-based design and the comprehensive data on almost all Danish adults and general practices. The individual-level information collected was obtained prospectively for all remunerated GP services and for demographic/socioeconomic characteristics using high-quality administrative health registers^25^^,^^26^ and long-term conditions from the Danish Multimorbidity Index.^28^ This made it possible to extensively control for confounding at the patient level. As GP services were incentivised, high completeness is expected for these data.^6^ The sampling approach accounted for random variation, even if all GPs had the exact same provision propensity. This allowed the authors to isolate and study the variation related to the chronic care profile of the individual practices.^36^

The study also had several limitations. The data on remunerated services were only available for the entire general practice. Thus, in practices with >1 GP, the mean service provision among the entire group of GPs was observed and the true variation may have been underestimated. Information on practice staffing was not available in the registers and may have a bearing on levels of service delivery. However, examining the service provision at practice level is a reasonable approach as GPs often distribute work across their team and share the same setting. It is up to the GP to initiate the service, whenever it is found relevant, as there is no central register of patients with chronic conditions qualifying for the services. Hence, the whole population is eligible for the services investigated in the study. Most long-term conditions that could justify chronic care service were included, but some may have been missed, which holds a risk of under-identification of long-term conditions. This under-identification is anticipated to be quite evenly distributed between practices after the comprehensive adjustment for patient characteristics, reducing the concern about systematic bias on the relative between-practices comparisons.

### Comparison with existing literature

To the authors’ knowledge, no other study has described the variation in chronic care services provided by GPs. Variation in healthcare utilisation has previously been studied,^20^^,^^37^ and several variation measures exist.^36^^–^39 Between-practice variation is commonly estimated through the coefficient of variation or using multilevel modelling.^40^^–^42 Yet, to estimate excess context-related variation, it must be taken into account that some variation is expected solely because of randomness.^30^

Variation may represent suboptimal treatment practices at either end of the utilisation spectrum (undertreatment or overtreatment).^17^ Identifying sources of potentially inappropriate variation is difficult because variation occurs at patient level, provider level, and healthcare system level.^17^ In the current study, extensive information at the patient level was used, which is known to account for the majority of the variation.^43^

Underuse of chronic care services among general practices with high proportions of patients with low socioeconomic status and multimorbidity may indicate social inequality in the provision of health care. This phenomenon has been described in the literature as the inverse care law, that is, the availability of good medical care tends to vary inversely with the need of the population served.^18^ The underlying reason for the low propensity to use chronic care services in some practices cannot be determined from the data in the current study. High workloads and GP burnout may impede the implementation of chronic care services.^44^ Notably, although the effect sizes of the associations related to practice propensities were small, they were still statistically significant for important patient characteristics.

### Implications for research and practice

The substantial variation found in the chronic care profiles of the included practices suggests that suboptimal treatment may occur, particularly in practices with populations characterised by high morbidity burden and socioeconomic deprivation. Despite a higher need, these practices provided chronic care to a relatively lesser extent. Although chronic care consultations were well remunerated, this economic incentive seems to be of limited benefit in counteracting these inverse care law mechanisms. Caution should be taken when comparing the specific service provision between different healthcare systems. Nevertheless, when such provider variation exists in a universal healthcare system, they are likely to exist in other healthcare systems, and this might increase inequality in health. Many countries are introducing incentives for chronic disease management, but simply incentivising such care may not benefit the patients at highest risk because the practices they attend may have less capacity to take on additional workload. Further research is needed to explore the causal explanations for variation in chronic care services and the implications for patient health outcomes such as mortality or hospital admissions. Such knowledge may improve understanding about the inverse care law and help design health systems that address health inequalities.^45^

In conclusion, chronic care was provided to the patients in most need of health care, that is, those with old age, multimorbidity, and low socioeconomic status. However, service provision varied across practices to an extent that could not be explained by differences in patient populations or by random variation. In practices with clustering of patients with multimorbidity and low socioeconomic status, the GPs provided fewer chronic care services than expected. The variation related to the chronic care profile of the practice might involve suboptimal chronic care delivery, which could suggest inverse care law mechanisms.
